# Scavenger Receptor Class A Plays a Central Role in Mediating Mortality and the Development of the Pro-Inflammatory Phenotype in Polymicrobial Sepsis

**DOI:** 10.1371/journal.ppat.1002967

**Published:** 2012-10-11

**Authors:** Tammy R. Ozment, Tuanzhu Ha, Kevin F. Breuel, Tiffany R. Ford, Donald A. Ferguson, John Kalbfleisch, John B. Schweitzer, Jim L. Kelley, Chuanfu Li, David L. Williams

**Affiliations:** 1 Department of Surgery, East Tennessee State University, Quillen College of Medicine, Johnson City, Tennessee, United States of America; 2 Department of Obstetrics and Gynecology, East Tennessee State University, Quillen College of Medicine, Johnson City, Tennessee, United States of America; 3 Department of Biomedical Sciences, East Tennessee State University, Quillen College of Medicine, Johnson City, Tennessee, United States of America; 4 Department of Medical Education, East Tennessee State University, Quillen College of Medicine, Johnson City, Tennessee, United States of America; 5 Department of Pathology, East Tennessee State University, Quillen College of Medicine, Johnson City, Tennessee, United States of America; 6 Department of Internal Medicine, East Tennessee State University, Quillen College of Medicine, Johnson City, Tennessee, United States of America; University of Michigan, United States of America

## Abstract

Sepsis is a frequent complication in critical illness. The mechanisms that are involved in initiation and propagation of the disease are not well understood. Scavenger receptor A (SRA) is a membrane receptor that binds multiple polyanions such as oxidized LDL and endotoxin. Recent studies suggest that SRA acts as a pattern recognition receptor in the innate immune response. The goal of the present study was to determine the role of SRA in polymicrobial sepsis. SRA deficient (SRA^−/−^) and C57BL/6JB/6J (WT) male mice were subjected to cecal ligation and puncture (CLP) to induce polymicrobial sepsis. NFκB activity, myeloperoxidase activity, and co-association of SRA with toll like receptor (TLR) 4 and TLR2 was analyzed in the lungs. Spleens were analyzed for apoptosis. Serum cytokines and chemokines were assayed. Blood and peritoneal fluid were cultured for aerobic and anaerobic bacterial burdens. Long term survival was significantly increased in SRA^−/−^ septic mice (53.6% vs. 3.6%, p<0.05) when compared to WT mice. NFκB activity was 45.5% lower in the lungs of SRA^−/−^ septic mice versus WT septic mice (p<0.05). Serum levels of interleukin (IL)-5, IL-6, IL-10 and monocyte chemoattractant protein −1 were significantly lower in septic SRA^−/−^ mice when compared to septic WT mice (p<0.05). We found that SRA immuno-precipitated with TLR4, but not TLR2, in the lungs of WT septic mice. We also found that septic SRA^−/−^ mice had lower bacterial burdens than WT septic mice. SRA deficiency had no effect on pulmonary neutrophil infiltration or splenocyte apoptosis during sepsis. We conclude that SRA plays a pivotal, and previously unknown, role in mediating the pathophysiology of sepsis/septic shock in a murine model of polymicrobial sepsis. Mechanistically, SRA interacts with TLR4 to enhance the development of the pro-inflammatory phenotype and mediate the morbidity and mortality of sepsis/septic shock.

## Author Summary

Trauma and other critical illnesses can progress to septic shock. The mechanisms that result in this progression are not understood. For this reason, there are no proven treatments available, and the mortality rate from sepsis remains quite high. We have found that mice that lack a certain cell surface protein, scavenger receptor A, have a higher rate of survival from a surgically induced sepsis than those that have the receptor. Previously, this receptor has been found to play a role in atherosclerosis, and more recently, to play a role in the immune response to infection. In this study we have found that in addition to improved survival, mice without scavenger receptor A have fewer bacteria in their abdominal cavities and in their blood. They also have lower levels of inflammation. We demonstrated that scavenger receptor A interacts with another protein involved in inflammation and infection, toll like receptor 4. This interaction might be one mechanism for the effects seen in mice without scavenger receptor A. These studies provide a better understanding of the underlying mechanisms of sepsis. Drugs that target scavenger receptor A could result in better therapies for sepsis.

## Introduction

The critically ill patient frequently develops a complex disease spectrum that may include acute respiratory distress syndrome, systemic inflammatory response syndrome, sepsis syndrome and/or septic shock [1]. Current wisdom implies that following severe injury or infectious challenge, the host responds by over-expressing inflammatory mediators resulting in a systemic inflammatory response that culminates in severe shock, multi-organ failure and death [2], [3], [4]. At present, we do not understand the cellular and molecular mechanisms that are involved in the initiation and propagation of septic injury; nor do we understand the physiologic mechanisms that attempt to maintain homeostasis and promote survival in the septic patient.

The macrophage scavenger receptor A (SRA, CD204, Entrez gene Msr1) is a type II membrane receptor [5]. SRA is primarily expressed by macrophages, though evidence suggests it may also be expressed by bone marrow derived and splenic dendritic cells [5]. SRA is a multi-functional receptor which binds endogenous ligands including oxidized LDL and apoptotic cells [5], [6], [7] and pathogen associated molecular patterns including endotoxin, lipoteichoic acid, and fungal glucans [7], [8], [9], [10], [6]. Evidence for direct intracellular signaling by SRA is limited and conflicting. However, several reports indicate that SRA interacts with Mer receptor tyrosine kinase [10], Lyn kinase [11] and PTK(Src)/Rac1/Jnk [12]. Additionally, phosphorylation of SRA may facilitate the interaction of the SRA transmembrane domain with signaling components [13]. It has also been reported that SRA induces activation of MyD88 dependent toll like receptor (TLR) 4 signaling and inhibits TLR4 dependent IRF3 activation in response to endotoxin or fucoidan [14]. Finally, it has been demonstrated that SRA interacts with TRAF6 upon exposure to endotoxin and prevents its degradation, thus limiting the inflammatory response to endotoxin [15]. When taken together, these data suggest that SRA does participate in intracellular signaling in response to ligand interaction.

SRA plays a role in several important pathological processes, including atherosclerosis [6]. SRA has also been identified as a pattern recognition receptor in the innate immune system [16] and thus is involved in the immune response to infectious disease [17], [18], [19], [20], [21], [22]. However, the role of SRA in response to infection is complex and not well understood. By way of example, SRA has been reported to be protective in *Listeria monocytogenes* infection [23], herpes simplex-1 infection [23], *Neisseria meningitides* septicemia [22] and pneumococcal pneumonia [20]. In contrast, SRA deficient mice challenged with *Pneumocystis carinii* infection cleared the organisms from the lung more efficiently when compared to wild type controls [21], suggesting that SRA contributes to the pathophysiology of *P. carinii* infection. However, these studies were all performed in single organism infections. The role of SRA in a clinically relevant model of polymicrobial sepsis has not been investigated. In the present study, we found that SRA plays a central and previously unknown role in mediating the pathophysiology of sepsis/septic shock in a murine model of polymicrobial sepsis. Specifically, SRA interacts with TLR4 thereby facilitating the development of the pro-inflammatory phenotype and mediating the morbidity and mortality of sepsis/septic shock.

## Results

### SRA deficient mice are significantly more resistant to polymicrobial sepsis and septic shock than are wild type mice

**Figure 1:**
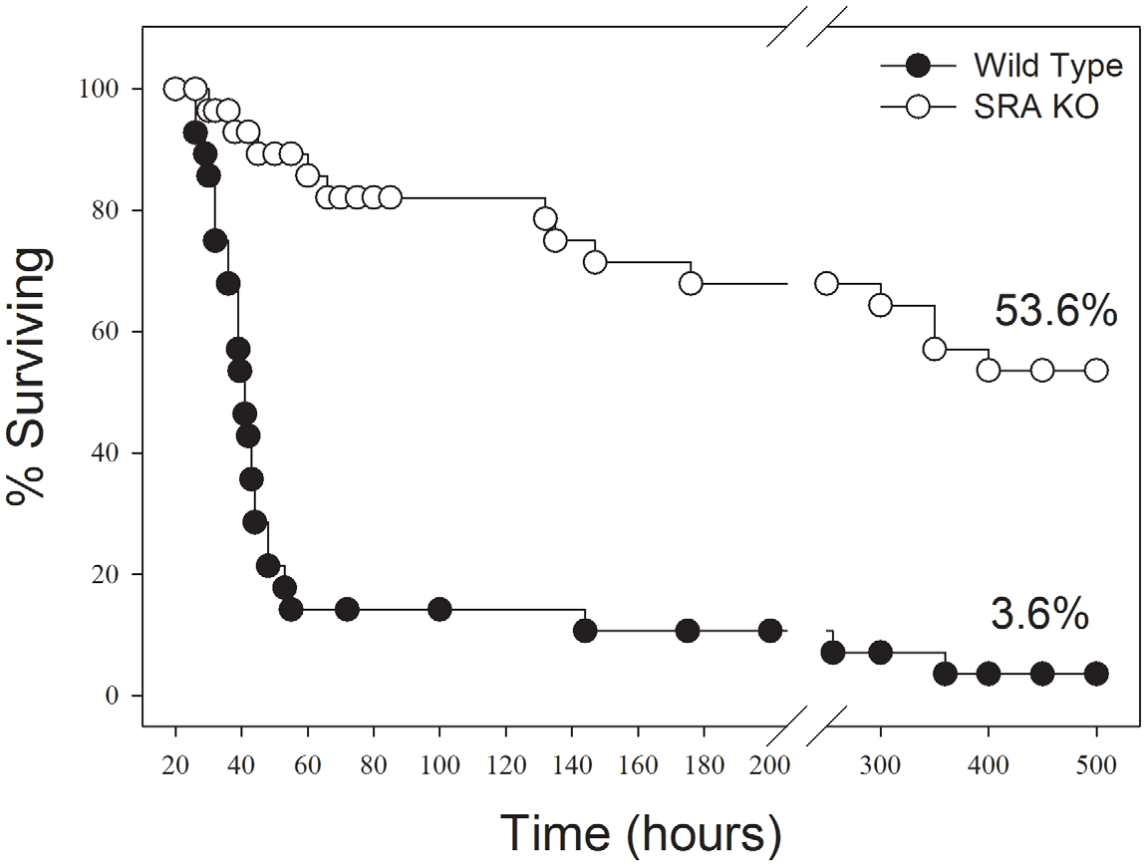
SRA deficient (SRA^−/−^) mice show increased long term survival in response to CLP induced sepsis. CLP was induced at time 0. The graph shows combined data from three replicate experiments. N = 28–30/group. p<0.01 SRA^−/−^ vs WT.

In response to CLP sepsis, we found that SRA^−/−^ mice showed a much longer median survival time (300 hrs vs 43 hrs) than did WT mice. Of greater importance, SRA deficient mice, with CLP sepsis, showed a significant increase in long term survival (53.6% vs. 3.6%, p<0.001) when compared to WT mice (**Figure 1**). That is to say that 53.6% of the SRA^−/−^ mice with sepsis went on to survive indefinitely while only 3.6% of the WT septic mice survived. These data strongly suggest that SRA contributes to the mortality associated with fulminating polymicrobial sepsis.

### Sepsis induced NF-κB activity is attenuated in the lungs of SRA deficient mice

**Figure 2:**
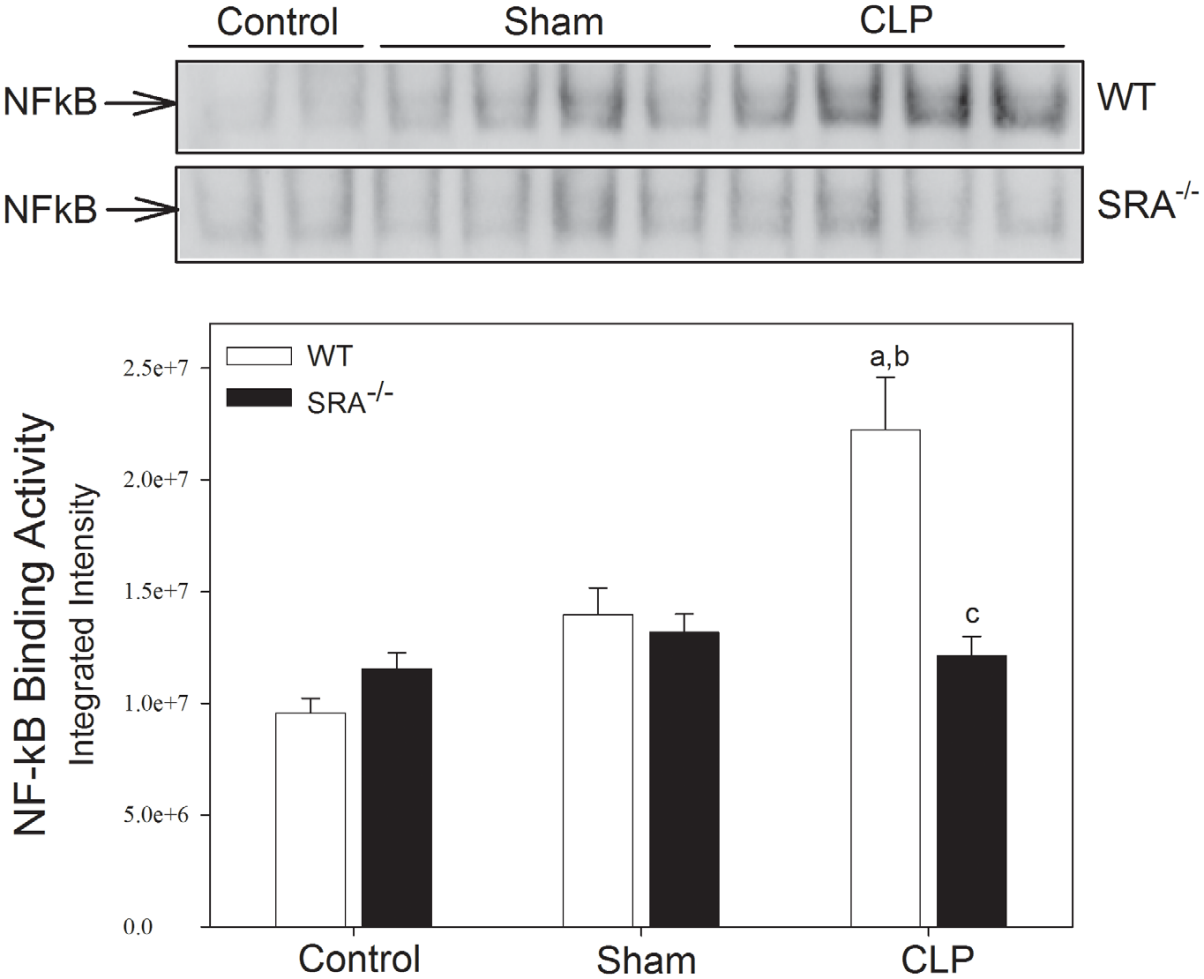
SRA^−/−^ mice show attenuated lung NFκB activity when compared to WT mice with CLP sepsis. Mice underwent CLP at time 0. Lung tissue was harvested 6 hrs after CLP. Pulmonary NFκB binding activity was measured by EMSA. N = 4/group; p<0.05 in: a vs. Control, b vs. Sham, and c vs. WT CLP.

To determine if differences in the inflammatory response were responsible for the differences in survival, NFκB activity was measured in WT and SRA^−/−^ mice in response to CLP by EMSA (**Figure 2**). In WT mice, CLP resulted in a significant increase in pulmonary NFκB activity (132.29% vs. WT control and 59.22% vs. WT sham; p<0.05). In contrast, NFκB activity was not significantly increased in the lungs of SRA^−/−^ mice with polymicrobial sepsis (14.12% and 5.05% vs. SRA^−/−^ control and sham mice, respectively; p>0.05), and was significantly less than that of the lungs from WT CLP mice (45.49%; p<0.05). These data illustrate that the inflammatory response to CLP induced sepsis is blunted in SRA^−/−^ mice.

### The increase in cytokine production in polymicrobial sepsis is attenuated in SRA deficient mice

**Figure 3:**
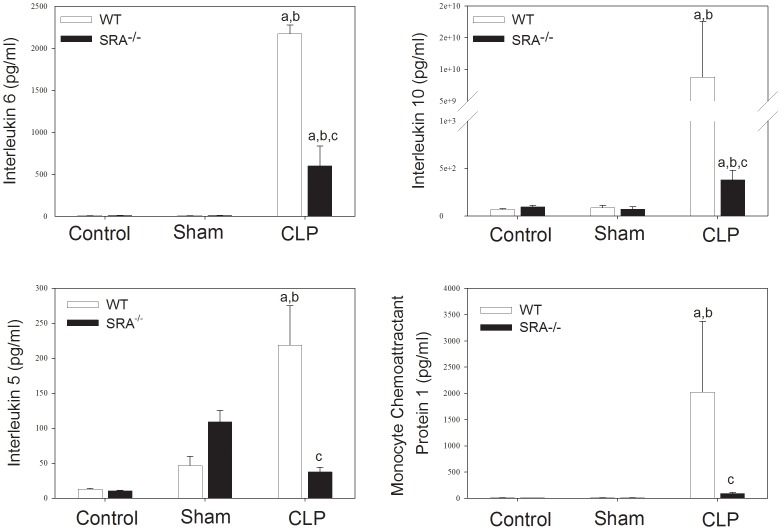
Systemic cytokine levels are attenuated in SRA^−/−^ when compared to wild type control mice. Sixteen hours post-operatively, mice were euthanized and serum cytokines were analyzed by Luminex. N = 6/group; p<0.001 in: a vs. Control, b vs. Sham, and c vs. WT CLP.

To further define the role of SRA in the inflammatory response in sepsis, serum cytokine levels were assayed in WT and SRA^−/−^ mice 16 hrs after CLP (**Figure 3**). CLP resulted in a significant increase in serum interleukin (IL)-6 (p<0.05) when compared to sham operated or control animals in both WT and SRA^−/−^ mice. However, IL-6 levels were decreased in SRA^−/−^ septic mice when compared to septic WT mice (↓72.4%; p<0.05). IL-10 was also increased in both WT and SRA^−/−^ septic animals compared to sham operated animals (p<0.05). Again, the increase in serum IL-10 was significantly blunted in septic SRA^−/−^ animals compared to WT CLP mice. (p<0.05). Sepsis resulted in a dramatic increase in monocyte chemoattractant protein-1 (MCP-1) when compared to sham control mice (**Figure 3**). Serum MCP-1 was also increased in SRA^−/−^ mice, when compared to sham (p<0.05), but the magnitude of the increase was significantly less than that observed in WT CLP mice. Specifically, serum MCP-1 levels were 95.4% less in septic SRA^−/−^ mice compared to septic WT mice (p<0.05). Finally, CLP resulted in a significant increase in circulating IL-5 levels in both WT (↑370%; p<0.05) and SRA^−/−^ (↑65.5%; p<0.05) mice compared to sham controls. As with the other cytokines, serum levels of the Th2 cytokine IL-5 were less in SRA^−/−^ CLP mice than in WT CLP mice (p<0.05). We did not detect significant differences in levels of the other cytokines assayed. These data demonstrate that the inflammatory phenotype, as viewed from the perspective of circulating cytokines and chemokines, is significantly attenuated in SRA^−/−^ mice. Additionally, the significant attenuation of Th2 associated cytokines in septic SRA^−/−^ mice indicates an overall maintenance of the Th1 phenotype in these mice.

### SRA deficiency has no effect on pulmonary neutrophil infiltration in polymicrobial sepsis

**Figure 4:**
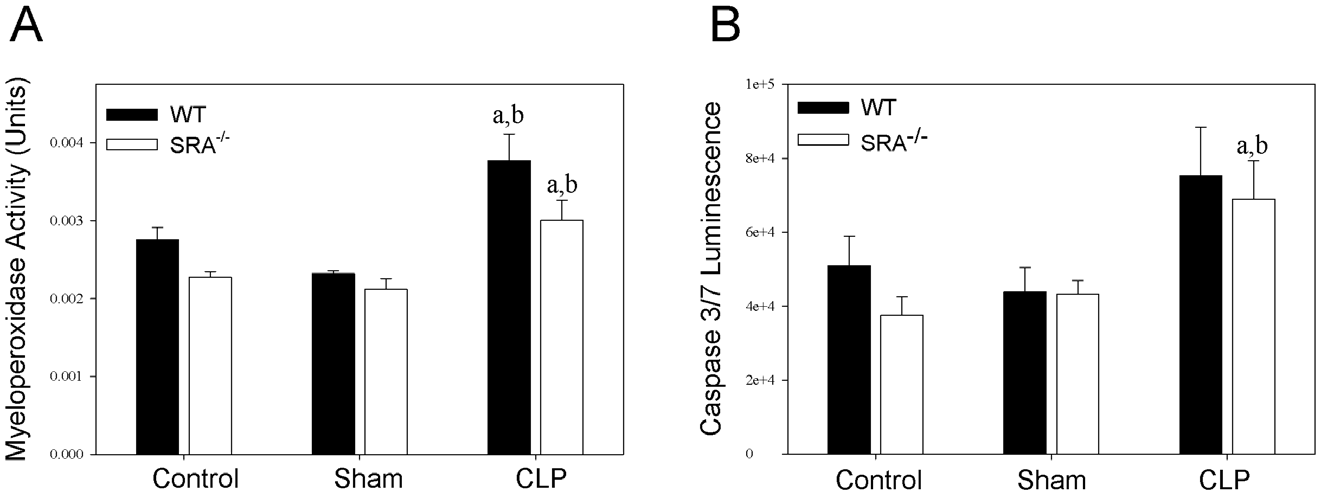
SRA deficiency had no effect on pulmonary neutrophil infiltration or splenocyte apoptosis during sepsis. Lung and spleen tissue were harvested at 16 hrs after CLP sepsis. Lungs were analyzed for myeloperoxidase activity (A), and spleens were analyzed for caspase 3/7 activity (B). N = 4/group. p<0.05 in: a vs. Control, b vs. Sham.

To further characterize the differences in the inflammatory response to sepsis in WT and SRA^−/−^ mice, neutrophil infiltration in the lung was determined by measuring myeloperoxidase (MPO) activity (**Figure 4**). Though there was a significant increase in MPO activity in CLP mice vs. sham mice, there was no difference between septic WT and SRA^−/−^ mice. To confirm these data, paraformaldehyde fixed lungs were sectioned and stained with hemotoxylin and eosin. The stained tissues were evaluated for inflammation and neutrophil infiltration by two independent pathologists. There was no difference detected in the lung tissues harvested from WT or SRA^−/−^ mice subjected to CLP (data not shown). Specifically, there was no evidence of increased neutrophils in the lungs, no foci of inflammation and no margination of neutrophils in blood vessels in any of the lung tissues examined (data not shown). These data indicate that SRA deficiency has no significant effect on pulmonary neutrophil infiltration or inflammation in sepsis.

### Lack of SRA has no effect on splenocyte apoptosis in polymicrobial sepsis

Apoptosis of splenic lymphocytes plays a role in the immunosuppression associated with sepsis [24]. To determine if there is a difference in splenocyte apoptosis in septic WT and SRA^−/−^ mice, spleens were lysed and caspase 3 and 7 activity was measured (**Figure 4**). Though there was a significant increase in caspase activity in septic SRA^−/−^ spleens compared to sham controls, there was no difference between WT sham and CLP nor was there a significant difference between WT and SRA^−/−^ in septic spleens.

### SRA associates with TLR4, but not TLR2, in the lungs of septic mice

**Figure 5:**
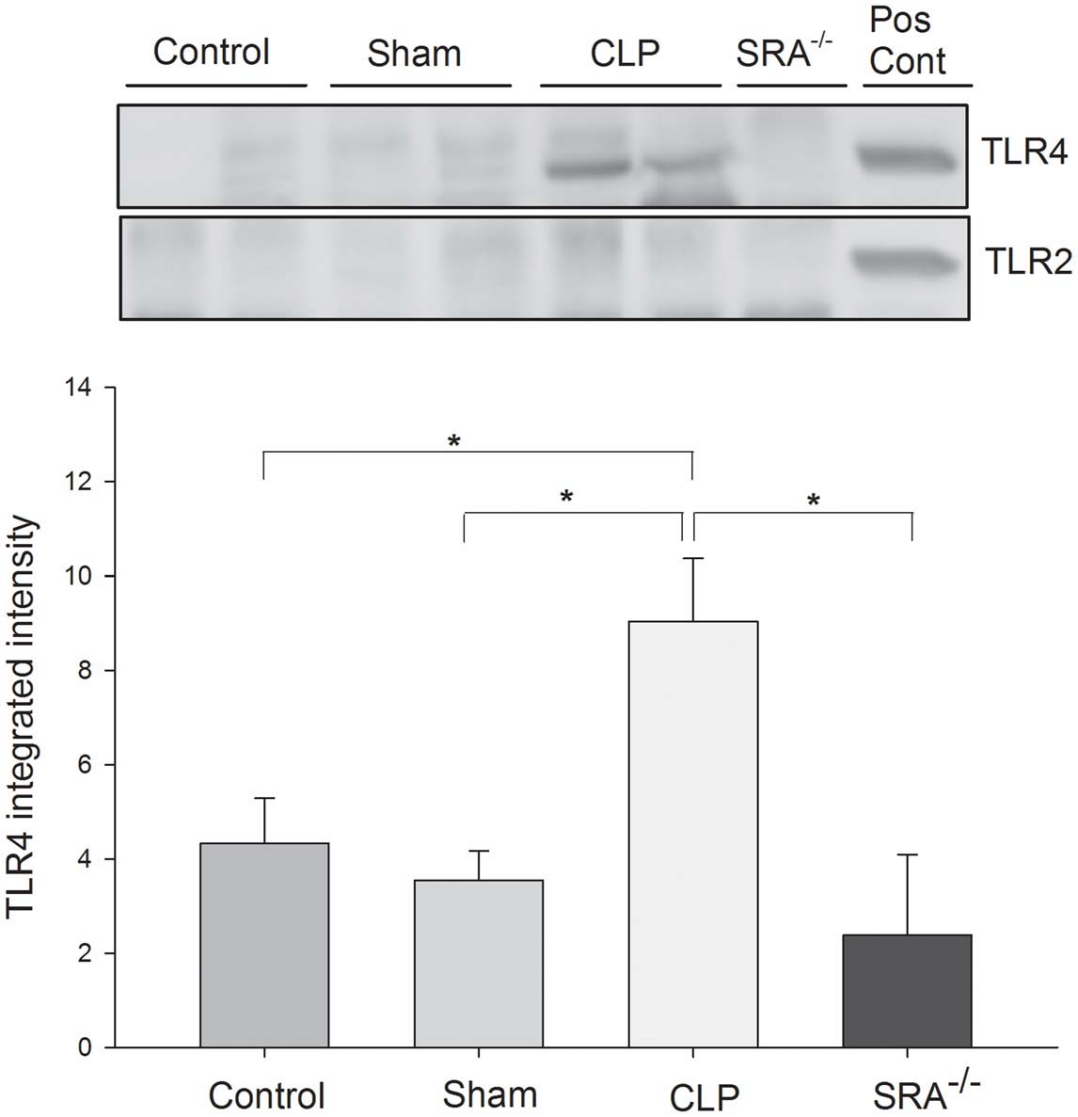
SRA co-associates with TLR4, but not TLR2, in lungs of wild type CLP mice. Lung tissue was harvested 3 hrs after CLP. Lysates were precipitated with anti-SRA and immunoblotted for TLR2 or TLR4. N = 4/group. * indicates p<0.05.

The data above indicate that SRA contributes to the inflammatory response in CLP sepsis, and that SRA deficiency correlates with improved survival. Since TLRs are known to play a major role in the inflammatory response to sepsis [25], [26], [27], we sought to determine if SRA interacts with TLRs during CLP sepsis. Protein from WT murine lungs was precipitated with SRA antibody and blotted for TLR2 and 4. We found that TLR2 was not precipitated with SRA in the control, sham, or CLP lungs (**Figure 5**). On the other hand, TLR4 was precipitated with SRA in CLP lungs and to a far lesser extent in control and sham lungs (**Figure 5**). These data indicate that SRA is exerting its inflammatory effect, in part, by interacting with TLR4, but not TLR2 in polymicrobial sepsis.

### SRA deficiency reduces overall bacterial burden and alters the bacterial population in septic mice

The previous experiments revealed that SRA deficiency results in decreased inflammation in polymicrobial sepsis. We sought to determine if SRA deficiency had an impact on bacterial burden and/or the composition of bacterial species post-CLP. Blood and peritoneal fluid were harvested from WT and SRA^−/−^ septic mice and cultured for aerobic and anaerobic bacteria. The bacterial burdens were quantified by quadrants positive for growth, and the species cultured were identified. One of four SRA^−/−^ showed no bacteria in the blood, while two others showed only non-pathogenic *Lactobacillus* (**Table 1**). The remaining SRA−/− blood sample also showed *Lactobacillus* as well as *E. coli* (**Table 1**). In contrast, all of the WT mice showed positive bacterial cultures (**Table1**). One contained only *Lactobacillus*, while the remaining three were culture positive for a pathogen, i.e. Group D *Enterococcus* or *Campylobacter gracilis* (**Table 1**). Culture of peritoneal fluid from WT and SRA^−/−^ mice showed a much greater number and diversity of micro-organisms (**Table 2**). Four of the five SRA^−/−^ mice had 1+ or less bacterial growth from their peritoneal fluid, and these bacteria were primarily non-pathogenic (**Table2** **)**. However, one mouse in this group did show *E. coli* in the blood and higher levels of peritoneal growth with 3+ E. coli and 2+ Staphylococcus (**Table 2**). As with the blood, WT mice showed greater levels of bacteria than SRA^−/−^ mice (**Table 2**). Each WT mouse had at least one bacterial species with 2+ growth, and all mice had at least 1+ growth of the pathogenic Group D *Enterococcus* in their peritoneal fluid. These data suggest that SRA plays a role in the clearance of bacteria in sepsis.

**Table 1:** Comparison of bacterial content and growth characteristics of blood from wild type and SRA^−/−^ wild mice 16 hours after CLP sepsis.

Mouse	Wild Type CLP	SRA^−/−^ CLP
1	Aerobes –	Aerobes – None
	Group D *Enterococcus Lactobacillus* sp.	Anaerobes – None
	Anaerobes - None	
2	Aerobes –	Aerobes –
	*Lactobacillus* sp.	*Lactobacillus* sp.
	Anerobes - None	Anaerobes – None
3	Aerobes – None	Aerobes –
	Anaerobes –	*Lactobacillus* sp.
	*Campylobacter gracilis*	Anaerobes - None
4	Aerobes –	Aerobes –
	Group D *Enterococcus*	*Lactobacillus* sp.
	*Lactobacillus* sp.	*E. coli*
	Anaerobes - None	Anaerobes - None

Blood was harvested aseptically from mice 16 h after CLP and cultured as described in the Methods section.

**Table 2:** Comparison of bacterial content and growth characteristics of peritoneal fluid from wild type and SRA^−/−^ mice 16 hours after CLP sepsis.

Mouse	Wild Type CLP	SRA^−/−^ CLP
1	Aerobes –	Aerobes –
	3+ a Group D *Enterococcus*	1+ *Lactobacillus* sp.
	3+ *Lactobacillus* sp.	Anaerobes –
	2+ Coagulase negative *Staphylococcus*	*Prevotella oris* (few colonies)^b^
	Anaerobes –	Gram negative coccobacillus (few colonies)^b^ *^,^* c
	3+ *Campylobacter gracilis*	
	3+ *Clostridium* sp.	
2	Aerobes –	Aerobes –
	3+ *Lactobacillus* sp.	1+ *Lactobacillus* sp.
	Anaerobes –	1+ *Streptococcus viridians*
	3+ *Campylobacter gracilis*	Anaerobes-
	3+ *Propionibacterium* sp.	1+ *Bacteroides distasonis*
	3+ Gram negative coccobacillus (few colonies)^b^ *^,^* c	1+ *Clostridium clostridioforme*
3	Aerobes –	Aerobes –
	2+ Group D *Enterococcus*	1+ *Lactobacillus* sp.
	2+ *Lactobacillus* sp.	Coagulase negative *Staphylococcus*
	Anaerobes – None	(few colonies)^b^
		1+ *Streptococcus viridans*
		Anaerobes – None
4	Aerobes –	Aerobes-
	1+ Group D *Enterococcus*	3+ E. coli
	2+ *Lactobacillus* sp.	2+ Coagulase negative *Staphylococcus*
	1+ Coagulase negative *Staphylococcus*	2+ *Lactobacillus* sp.
	Anaerobes –	Anaerobes –
	3+ Gram negative rod (unidentified)^c^	1+ *Prevotella oris*
		1+ *Bacteroides distasonis*
		2+ Gram negative rod (unidentified)^c^
5	Aerobes-	Aerobes –
	3+ Group D *Enterococcus*	1+ *Streptococcus viridans*
	1+ *Streptococcus viridians*	1+ *Lactobacillus* sp.
	Anerobes- None	Anaerobes –
		1+ *Bacteroides distasonis*

Peritoneal fluid was harvested aseptically from mice 16 h after CLP and cultured as described in the Methods section.

a Growth was estimated by scoring the number of plate quadrants covered with colonies following streaking, *i.e.* 1+ = one quadrant, 2+ = two quadrants, etc.

b Only isolated colonies were observed that did not fill one quadrant.

c Definitive identification of the genus and species was not possible. In this case, bacteria were identified based solely on Gram stain morphology and growth characteristics.

## Discussion

Several important observations emerged from this study. Our data indicate that SRA deficient mice are much more resistant to fulminating polymicrobial sepsis, as demonstrated by increased long term survival. In association with the improved survival, SRA deficient mice showed an attenuated inflammatory phenotype as determined by decreased organ NFκB activity and attenuation of sepsis induced serum cytokine levels. In addition, SRA^−/−^ mice showed lower bacterial burdens and fewer pathogenic bacteria when compared to WT mice. In order to elucidate the mechanisms by which SRA facilitates the morbidity and mortality of sepsis, we discovered that SRA co-associates with TLR4 during sepsis and that this interaction is closely correlated with tissue NFκB activation, development of a pro-inflammatory phenotype and mortality in sepsis. When considered as a whole, our data suggest that SRA plays a key role in the morbidity and mortality of sepsis via its interaction with TLR4.

In the present study, SRA deficiency resulted in a significant attenuation of sepsis-induced NFκB activation, which correlated with improved survival outcome. Whether this is a cause-and-effect relationship cannot be established by the present data, but we and others have shown that NFκB dependent signaling plays a major role in the morbidity and mortality of CLP sepsis [28], [29], [30]. How SRA contributes to NFκB activation is not clear, since the ability of SRA to transduce an intracellular signal remains controversial [31], [12], [10]. SRA lacks a signaling motif in the cytoplasmic tail suggesting that it does not signal [31], though treatment of cells with known SRA ligands does result in signal transduction [12]. One explanation for these data would be that SRA interacts with other receptors that do have the ability to induce signal transduction. Indeed, our data support this explanation by demonstrating that SRA interacts with TLR4 in CLP sepsis. TLR4 is known to play an important role in septic inflammation and is a well known inducer of NFκB activity and inflammatory cytokine production [25], [26], [27]. Our data suggest that the interaction of SRA with TLR4 amplifies the signal generated by TLR4 in response to the bacterial and endogenous ligands released during polymicrobial sepsis. Loss of the interaction with SRA would then result in loss of the amplification and a lower overall inflammatory response. In fact, we demonstrated that sepsis-induced cytokine/chemokine expression was attenuated in SRA deficient mice. Of specific interest, both IL-6 and IL-10 levels, which strongly correlate with survival outcome in sepsis [32], [33], were significantly attenuated in SRA deficiency. When taken together these data suggest that in response to sepsis, SRA interacts and/or cooperates with TLR4 to enhance NFκB activation and cytokine/chemokine expression with a concomitant increase in inflammatory phenotype and mortality. Thus, our data indicate that SRA is required for a maximal TLR4/NFκB response to CLP sepsis. These data are consistent with previous reports which indicate that SRA can function as a co-receptor [14].

Interestingly, SRA does not appear to play a role in all aspects of the response to CLP sepsis. Lung neutrophil sequestration and splenocyte apoptosis have been implicated in the pathophysiology of sepsis [34], [35]. Tissue neutrophil infiltration, adhesion and degranulation are thought to play a prominent role in tissue damage during sepsis [34]. Surprisingly, we found that SRA does not appear to play a significant role in sepsis induced pulmonary neutrophil infiltration and sequestration, despite differences in circulating inflammatory cytokines. It is possible that pulmonary neutrophil infiltration/inflammation does not play a significant role in survival outcome in our model of acute sepsis. Splenocyte apoptosis is thought to play a central role in late immune dysfunction in sepsis and may contribute to sepsis associated multiple organ failure [35]. However, we did not detect any significant difference in splenic apoptosis between WT and SRA^−/−^ mice in response to sepsis. Lymphocytes are a major portion of the splenic leukocyte population; therefore these data primarily reflect apoptosis in lymphocytes. Why SRA deficiency would not have an effect on lymphocyte apoptosis while still improving survival outcome is unclear. It may be that splenocyte apoptosis does not play a significant role in the pathophysiology in this model of acute sepsis, but may contribute to later septic sequelae.

We also observed that SRA deficiency correlated with decreased bacterial burden in response to CLP sepsis. This was particularly true for the peritoneal cavity. It is not clear why SRA^−/−^ mice would have a decreased bacterial burden in response to CLP sepsis. Decreased bacterial burden in septic SRA^−/−^ septic mice might seem counterintuitive since SRA is known to facilitate the uptake of bacterial products such as LPS and LTA, and it has been reported to mediate the non-opsonic phagocytosis of bacteria [36], [20], [8], [22]. Indeed, SRA deficient mice have been reported to show higher levels of bacteremia in *Listeria monocyotgenes and Neisseria meningitides* septicemia when compared to WT mice [22]. However, in *Pneumocystis carinii* infection, SRA deficient mice cleared the organisms from the lung more efficiently than wild type controls [21]. These data suggest that the response of SRA to infection is complex and may be dependent on the pathogen(s) encountered. In the case of a polymicrobial infection such as CLP sepsis, it appears that SRA contributes to bacterial burden, particularly in the peritoneal cavity. The mechanisms responsible are unclear, but one possible mechanism is that SRA^−/−^ mouse macrophages and/or neutrophils are better able to kill microbes, perhaps due to an overall maintenance of the Th1 immune response. Future studies are warranted to determine how SRA deficient mice are better able to clear the bacteria associated with polymicrobial sepsis.

This is the first report documenting the role of SRA in polymicrobial sepsis. In 2005, Cotena and colleagues reported on the role of SRA in a sterile peritonitis model which employed the injection of zymosan to elicit peritoneal inflammation [37]. These investigators reported that SRA is a non-activating receptor that serves to counter the activities of pro-inflammatory receptors and attenuates the production of specific chemokines to ensure an inflammatory response of the appropriate magnitude [37]. However, Cotena et al did not study the role of SRA in a model of infection, such as CLP [37], so comparisons between our results and theirs must be made with caution. Kobayashi [17], Chen [38] and colleagues have reported that SRA deficient mice are more resistant to LPS challenge, suggesting that SRA plays a role in endotoxic shock. However, Yu et al have recently reported that SRA attenuates TLR4 induced NFκB activation in an endotoxemia model by directly inhibiting ubiquitination of TRAF6 [15]. In addition, Yu et al reported that SRA^−/−^ mice are more susceptible to endotoxin challenge [15]. The reasons for the differences between the work of Kobayashi [17], Chen [38] and colleagues and Yu et al [15] are not readily apparent. However, it is important to note that treatment of cultured cells with endotoxin, or injection of animals with endotoxin, is not a reliable surrogate for polymicrobial sepsis/septic shock [3]. Indeed, numerous reports have clearly delineated the differences between endotoxemia and a clinically relevant *in vivo* model of sepsis, such as CLP [3], [39], [40], [41]. Consequently, the effect of SRA in endotoxemia may not be indicative of the role that SRA plays in a fulminating polymicrobial sepsis model, such as CLP, or in clinical sepsis for that matter [40], [41]. We believe that by examining SRA in a clinically relevant model of sepsis, such as CLP, we will have a more accurate assessment of the role of this receptor in septic disease.

In conclusion, our data indicate that the scavenger receptor class A plays a key role in mediating the pathophysiology of fulminant sepsis/septic shock. To the best of our knowledge, this is the first report documenting the deleterious effects of SRA in a clinically relevant model of polymicrobial sepsis. Specifically, SRA appears to be necessary for maximal development of the pro-inflammatory phenotype, in part, through interaction and co-operativity of SRA with the TLR4/NFκB signaling pathway. These data advance our knowledge of the *in vivo* mechanisms of sepsis and, of potentially greater importance, suggest that modulation of SRA activity may be a viable approach to the management of sepsis syndrome.

## Materials and Methods

### Ethics statement

All animal procedures were conducted in strict compliance with the National Institutes of Health “Guide for the Care and Use of Laboratory Animals”. The animal protocol was reviewed and approved (protocol number 101201) by the University Committee on Animal Care at the James H. Quillen College of Medicine, East Tennessee State University under the guidelines of the Association for Assessment and Accreditation of Laboratory Animal Care, US Department of Agriculture, and the Public Health Service guidelines for the care and use of animals as attested by the National Institutes of Health. All efforts were made to minimize suffering.

### Mice

The SRA knock-out (SRA^−/−^) mouse was originally generated by Suzuki et al [23]. Breeding pairs of SRA^−/−^ mice were kindly provided by Siamon Gordon, University of Oxford. WT control mice, C57BL/6J, were purchased from Jackson Labs (Bar Harbor, ME). The animals were maintained on standard laboratory chow and water *ad libitum* with a 12-hour light/dark cycle. Serologic testing confirmed that the mice were virus free.

### Polymicrobial sepsis

Age and weight matched male mice underwent cecal ligation and puncture (CLP) as previously described to induce polymicrobial sepsis [28], [42], [43]. Surgery was performed under isoflurane anesthesia. Briefly, the cecum was exteriorized, the contents were massaged distally, and the cecum was ligated distal to the ileocecal junction. The cecum was punctured once with a 20 gauge needle in an avascular region near the distal end, and a bleb of cecal contents was extruded from the puncture. Sham surgery (laparotomy alone) mice were used as a control for surgery and anesthesia, and animals that underwent no surgery or anesthesia were employed as negative controls. Mice were sacrificed at 16 hr post-operatively. Lungs, spleens, and sera were harvested, flash frozen, and stored in liquid nitrogen. Blood and peritoneal fluid were harvested and immediately cultured for bacterial growth. Parallel groups were followed for survival. Mice were terminated upon becoming moribund.

### Immunoprecipitation

Approximately 1 mg of lung cellular proteins were immunoprecipitated with 2 µg of antibodies to SRA (Santa Cruz Biotechnology, Santa Cruz, CA) for 1 h at 4°C followed by the addition of 15 µl of protein A/G-agarose beads (Santa Cruz Biotechnology) as previously described [44]. The precipitates were washed four times with lysis buffer and subjected to immunoblotting with the appropriate antibodies.

### Western blot

Precipitated pulmonary proteins were immunoblotted as described previously [45], [46]. Briefly, the proteins were separated by SDS-polyacrylamide gel electrophoresis and transferred onto Hybond ECL membranes (Amersham Pharmacia, Piscataway, NJ). The ECL membranes were incubated with anti-TLR4 or TLR2 (Santa Cruz Biotechnology), followed by incubation with peroxidase-conjugated secondary antibodies (Cell Signaling Technology, Danvers, MA). The signals were detected with the ECL system (GE Healthcare, Piscataway, NJ). To control for lane loading, the same membranes were probed with anti-GAPDH (glyceraldehyde-3-phosphate dehydrogenase, Biodesign, Saco, Maine) after being washed with stripping buffer. The signals were quantified by scanning densitometry using a Bio-Image Analysis System (Bio-Rad, Hercules, CA).

### Electrophoretic mobility shift assay (EMSA)

Nuclear proteins were isolated from lung samples as previously described [45], [46]. NFκB binding activity was examined by EMSA in a 15 µl binding reaction mixture containing 15 µg of nuclear proteins and 35 fmols of [γ-^32^P] labeled double-stranded NFκB consensus oligonucleotide.

### Pulmonary myeloperoxidase activity

Tissues were processed as directed by the MPO Fluorometric Detection Kit (Assay Designs, Ann Arbor, MI). Specifically, 50 mg of tissue was weighed out into 1× assay buffer containing 10 mM N-ethylmaleimide (Sigma). The samples were homogenized by using a Polytron homogenizer. After pelleting, the cells were lysed using 0.5% hexadecyltrimethylammonium in 1× assay buffer. Following homogenization by Polytron and sonication at 50% power for 3–10 s pulses, the homogenates were subjected to two freeze thaw cycles. After clearing of cell debris by centrifugation, the lysates were stored at −80°C until assayed. The samples were assayed according to kit directions, and the fluorescence was measured using the Modulus Microplate fluorescent plate reader after 30 min incubation (Turner Biosystems, Sunnyvale, CA).

### Pulmonary histology

The lungs were fixed in formalin, put into paraffin by an automated tissue processor, cut at 8 µm and stained with hematoxylin and eosin by standard methods. The resultant tissue sections were examined by two pathologists.

### Serum cytokines

Serum cytokine levels were assayed with an Invitrogen murine 20 plex cytokine assay (Carlsbad, CA) on a Luminex 100 instrument. Specifically, we assayed the serum for FGF basic, GM-CSF, IFN-γ, IL-1α, IL-1β, IL-2, IL-4, IL-5, IL-6, IL-10, IL-2p40/p70, IL-13, IL-17, IP-10, KC, MCP-1, MIP-1α, MIG, TNFα and VEGF. Cytokine levels were established by comparison to a standard curve as per the manufacturer's instructions.

### Spleen apoptosis

Caspase 3 and 7 activities were measured in splenic lysates using the Caspase-Glo 3/7 Assay from Promega according to manufacturer's directions. Briefly, whole spleens were disrupted in TBS with a Polytron homogenizer. After centrifugation, the pellets were suspended in hypotonic lysis buffer (10 mM HEPES, 10 mM KCL, 0.1 Mm EDTA, 0.1 mM EGTA, and protease inhibitors) and homogenized using a Polytron. After incubation on ice for 1 h, 10% NP-40 was added and the lysates were vortexed at high speed for 1 min. The insoluble fraction was removed by centrifugation, and the protein concentration of the lysates was measured by BCA. The protein concentration was adjusted to 10 µg/ml, and the lysates were mixed with an equal volume of kit reagent. Luminescence was measured at 2 h of incubation with a Modulus Microplate reader.

### Bacteriology

Peritoneal fluid and blood (∼300 µl) were harvested aseptically from mice 16 h after CLP. Blood samples were inoculated into fresh thioglycollate broth (6 ml) and incubated at 37°C for 48 hr at which time 0.025 ml aliquot of broth was streaked to produce isolated colonies on 5% sheep blood agar plates. An anaerobic blood agar plate was also streaked and incubated. In parallel, two 0.025 ml of peritoneal fluid was directly streaked and incubated in the same manner. Blood plates were incubated in a candle extinction jar at 37°C for 72 hr. The anaerobic blood agar plates were incubated in an anaerobe jar (BD Gas Pak EZ Anaerobe Container System, BD Biosciences, San Diego, CA) at the same temperature for a comparable amount of time. Growth was estimated by scoring the number of plate quadrants covered with colonies, i.e. 1+ = one quadrant, 2+ = two quadrants, etc. Isolated colonies were separated by colony morphology and Gram stained. Each colony was purified and subjected to differential tests including oxidase, catalase, indol, etc. as appropriate for their Gram stain morphology. Aerobes were identified by standard methods.

### Statistical analysis

Survival trends (**Figure 1**) were plotted with Kaplan-Meier technique and compared with the log-rank test. Continuous measurements of study groups were summarized with the mean and sem (**Figures 2** **–** **5**); group mean levels were compared with the 1-way analysis of variance followed by the least significant difference comparison strategy. A probability level of 0.05 or smaller was used to indicate statistical significance.
